# A five-long non-coding RNA signature to improve prognosis prediction of clear cell renal cell carcinoma

**DOI:** 10.18632/oncotarget.17506

**Published:** 2017-04-28

**Authors:** Da Shi, Qinghua Qu, Qimeng Chang, Yilin Wang, Yaping Gui, Dong Dong

**Affiliations:** ^1^ Shanghai Key Laboratory of Regulatory Biology, Institute of Biomedical Sciences, School of Life Sciences, East China Normal University, Shanghai, China; ^2^ Department of Urology, Pudong People's Hospital, Shanghai, China; ^3^ Department of Urology, Tongji Hospital, Tongji University School of Medicine, Shanghai, China; ^4^ Department of General Surgery, Minhang Hospital, Fudan University, Shanghai, China; ^5^ Department of Hepatic Surgery, Fudan University Shanghai Cancer Center, Shanghai, China

**Keywords:** long non-coding RNA, clear cell renal cell carcinoma, prognosis

## Abstract

Recent works have reported that long non-coding RNAs (lncRNAs) play critical roles in tumorigenesis and prognosis of cancers, suggesting the potential utility of lncRNAs as cancer prognostic markers. However, lncRNA signatures in predicting the survival of patients with clear cell renal cell carcinoma (ccRCC) remain unknown. In this study, we attempted to identify lncRNA signatures and their prognostic values in ccRCC. Using lncRNA expression profiling data in 440 ccRCC tumors from The Cancer Genome Atlas (TCGA) data, a five-lncRNA signature (*AC069513.4*, *AC003092.1*, *CTC-205M6.2*, *RP11-507K2.3*, *U91328.21*) has been identified to be significantly associated with ccRCC patients’ overall survival in both training set and testing set. Based on the lncRNA signature, ccRCC patients could be divided into high-risk and low-risk group with significantly different survival rate. Further multivariable Cox regression analysis suggested that the prognostic value of this signature was independent of clinical factors. Functional enrichment analyses showed the potential functional roles of the five prognostic lncRNAs in ccRCC oncogenesis. These results indicated that this five-lncRNA signature could be used as an independent prognostic biomarker in the prediction of ccRCC patients’ survival.

## INTRODUCTION

Renal cell carcinoma (RCC) is one of the most common renal malignancies worldwide, with an estimated 15,000 deaths every year [1]. Recent studies showed that incidence and mortality rates of RCC are increasing in the United States [2]. The vast majority of RCC subtypes are classified as clear cell renal cell carcinoma (ccRCC), which account for 70–80% of all RCCs. ccRCC has been reported to have the highest rate of progression and mortality [3, 4]. The standard of care for ccRCC remains surgical excision, and many ccRCC patients will be cured by surgery. However, about 30% of ccRCC patients had metastases and would die following removal of a confined tumor [4, 5]. To date, no widely accepted molecular biomarkers for ccRCC aggressiveness have been available. Great efforts to improve the early-stage detection of ccRCC are warranted.

Long non-coding RNAs (lncRNAs) are defined as RNA transcripts longer than 200 bp with little or no protein-coding capacity [6–9]. Mounting evidence suggested that lncRNAs are important molecular players with the ability of regulating gene expression at the level of chromatin modification, transcription and post transcriptional regulation [10–13]. Many dysregulated lncRNAs have been identified in cancers, which are tightly associated with tumor metastasis [14–17]. Moreover, many lncRNAs have been reported to be novel survival predictors for cancer patients, providing a broaden option for cancer diagnosis and prognosis [18–21]. Several prognostic biomarkers for ccRCC have been reported in clinical trials, such as *Linc00152* [22] and *lnc-ZNF180-2* [23], etc. More potential and valuable lncRNA biomarkers are needed to be identified to improve the clinical outcome of ccRCC patients [24–26].

Prognostic lncRNA signatures have been examined in many cancer types [18, 27–31]. In this work, we used a cohort of 440 ccRCC patients from The Cancer Genome Atlas (TCGA) data to detect the potential lncRNA signature in predicting the survival of ccRCC patients. We identified a five-lncRNA signature from the TCGA dataset, and determined its independence of clinical factors. The identification of prognostic lncRNAs suggested the potential roles of lncRNA in ccRCC pathogenesis.

## RESULTS

### Detecting the prognostic lncRNAs from the training set

The 440 TCGA ccRCC patients were randomly divided into a training (n = 220) set or a testing set (n = 220), respectively. Based on the training set, the lncRNAs were subjected to univariable Cox regression model, and a total of five lncRNAs were significantly correlated with the patients overall survival (*P-value* < 0.001; Table 1). Three of them (*AC069513.4*, *AC003092.1*, *RP11-507K2.3*) had positive coefficients, representing that the higher expression level was associated with shorter survival. The negative coefficients for the remaining two lncRNAs (*CTC-205M6.2*, *U91328.21*) suggest higher levels of expression were related to longer survival.

**Table 1:** Five lncRNAs significantly associated with the overall survival

Gene ID	Gene symbol	*P-value*	Hazard ratio	Coefficient
ENSG00000229178	*AC069513.4*	2.31e-06	4.72	1.43
ENSG00000236453	*AC003092.1*	1.96e-05	4.26	0.81
ENSG00000245060	*CTC-205M6.2*	3.31e-08	-5.52	-6.56
ENSG00000258789	*RP11-507K2.3*	3.76e-05	4.12	1.64
ENSG00000272558	*U91328.21*	2.78e-06	-4.68	-1.72

### The five-lncRNA signature and patients’ survival in the training set

**Figure 1:**
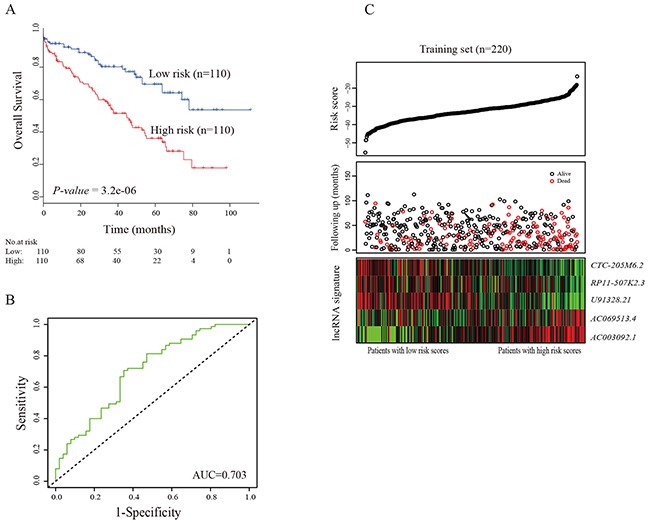
The five-lncRNA related risk score model predicts overall survival of patients with ccRCC in the training set (**A**) Kaplan-Meier estimates plots of the survival of the ccRCC patients with high- and low-risk groups. The *P-value* represents the differences among the two curves from the results of two-sided log-rank tests. The number below the curve represent the number of the patients in the high- and low-risk groups; (**B**) The Receiver operating characteristic (ROC analysis of risk score for survival prediction in the training set. The area under the curve (AUC) was calculated for ROC curves, and sensitivity and specificity were calculated to assess the score performance. (**C**) The five-lncRNA based risk score distribution, patients’ survival status and heatmap of the five lncRNA expression profiles.

Base on the expression level of five lncRNAs, we designed a risk-score formula for ccRCC patients’ survival prediction. The risk score formula is as following: Risk score= (1.43 × expression level of *AC069513.4*) + (0.81 × expression level of *AC003092.1*) + (1.64 × expression level of *RP11-507K2.3*) + (-6.56 × expression level of *CTC-205M6.2*) + (-1.72 × expression level of *U91328.21*). Next, we calculated the lncRNA-based risk score for each ccRCC patient in the training set, and divided ccRCC patients into high-risk (n=110) and low-risk groups (n=110) using the median risk score value as a threshold. The Kaplan-Meier curves showed that patients in the high-risk group suffered worse prognosis than the patients in the low-risk group (33.3 months vs. 40.1 months, *P-value*=3.2e-6; Figure 1A). The overall survival rate of the patients in the low-risk group was 80% in 3 years, 70% in 6 years and 60% in 9 years, whereas the survival rate in high-risk group was only 65% in 3 years, 45% in 6 years and 20% in 9 years, respectively. To evaluate the competitive performance of the five-lncRNA signature, time-dependent ROC curve analysis was measured, and the AUC score for the five-lncRNA signature was 0.703 (Figure 1B), demonstrating the better performance of survival prediction in the training dataset. Univariate Cox regression analysis showed that the five-lncRNA risk score were significantly associated with patients’ survival (*P-value* < 0.001, HR = 1.151, 95% CI = 1.1-1.2; Table 2). The distribution of the risk score, overall survival and expression profiles of five lncRNAs in samples of the training dataset were showed in Figure 1C, which were ranked according to the risk score value. Patients with high-risk scores had higher mortality than patients with low-risk scores. For patients with high risk scores, the expression profiles of lncRNAs (*AC069513.4*, *AC003092.1* and *RP11-507K2.3*) were significantly up-regulated, whereas the remaining two lncRNAs (*CTC-205M6.2* and *U91328.21*) were down-regulated.

**Table 2:** Univariable and multivariable Cox regression analyses in each data set

Variables	Univariable model^a^			Multivariable model		
	HR	95% CI of HR	P value	HR	95% CI of HR	P value
**Training set (n=220)**						
Five-lncRNA risk score	1.151	1.104-1.200	<0.001	1.131	1.086-1.178	<0.001
Age	1.019	1.001-1.038	0.034	1.021	1.002-1.040	0.032
Gender	1.016	0.654-1.580	0.943	0.780	0.491-1.238	0.291
AJCC stage	3.675	2.323-5.816	<0.001	3.390	2.104-5.449	<0.001
Tumor grade	2.820	1.718-4.630	<0.001	1.752	0.999-3.071	0.050
Testing set (n=220)						
Five-lncRNA risk score	1.057	1.011-1.104	0.014	1.055	1.014-1.098	<0.001
Age	1.040	1.017-1.063	<0.001	1.041	1.017-1.066	<0.001
Gender	0.904	0.540-1.513	0.701	1.339	0.777-2.309	0.294
AJCC stage	5.121	2.908-9.016	<0.001	4.440	2.419-8.150	<0.001
Tumor grade	2.374	1.326-4.252	0.004	1.542	0.834-2.852	0.167
Entire set (n=440)						
Five-lncRNA risk score	1.106	1.073-1.139	<0.001	1.093	1.063-1.125	<0.001
Age	1.029	1.015-1.044	<0.001	1.030	1.015-1.045	<0.001
Gender	0.958	0.685-1.339	0.802	0.964	0.678-1.371	0.179
AJCC stage	4.217	2.956-6.015	<0.001	3.269	2.232-4.787	<0.001
Tumor grade	2.582	1.770-3.767	<0.001	1.675	1.121-2.501	0.012

^a^ In both univariable and multivariable Cox regression analyses, risk score, age, gender, AJCC stage and Tumor grade were evaluated as continuous variables. P<0.05 was considered statistically significant in all analyses.

### Validation of the five-lncRNA signature for the survival prediction in testing set and the entire TCGA data set

**Figure 2:**
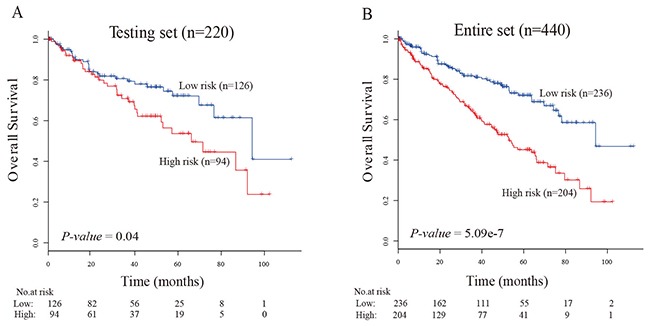
The five-lncRNA related risk score model predicts overall survival of patients with ccRCC in the testing set and the entire set (**A**) Kaplan-Meier estimates plots of the survival of the ccRCC patients using the five-lncRNA signature-related risk score model in the testing set (n=220). (**B**) Kaplan-Meier estimates plots of the survival of the ccRCC patients using the five-lncRNA signature-related risk score model in the entire set (n=440).

We next validated our five-lncRNA signature in the testing set to confirm our findings. By calculating the risk score for each patient in the testing set based on the same risk score formula, we divided ccRCC patients into a high-risk group (n=94) and a low-risk group (n=126) using the same threshold. Consistent with the results in the training set, patients in the high-risk group had significantly shorter survival than those in the low-risk group (33.07 months vs. 36.55 months, log-rank test *P-value*=0.04; Figure 2A). The overall survival rate of the patients in the low-risk group was 55% in 3 years, 12% in 6 years and 2% in 9 years, whereas the survival rate in high-risk group was only 48% in 3 years, 8% in 6 years and 0% in 9 years, respectively. In the entire TCGA data set, similar result was observed that patients in the high-risk group had significantly shorter survival than those in the low-risk group (33.4 months vs. 37.4 months, *P-value*=5.09e-7; Figure 2B). Time dependent ROC curves analysis for the five-lncRNAs signature-based model achieved AUC score of 0.63 and 0.68 in the testing set and entire set, respectively.

### Independence of the five-lncRNA signature and the other clinical variables

**Figure 3:**
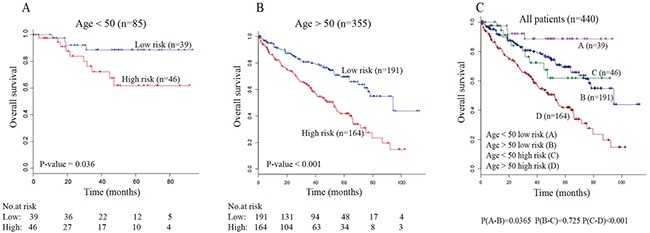
Stratification analyses of all patients adjusted to age using the five-lncRNA signature (**A**) The Kaplan-Meier plot of the younger patients with ccRCC (age < 50, n=85). (**B**) The Kaplan-Meier plot of the elder patients with ccRCC (age > 50, n=355). (**C**) The Kaplan-Meier plot of the entire patients with ccRCC (N=440). The number below the curve represents the number of the patients in the high- and low-risk group. The *P-value* represents the differences between the two curves from the results of two-sided log-rank tests.

**Figure 4:**
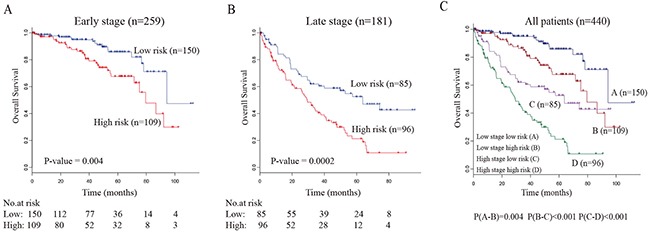
Stratification analyses of all patients adjusted to the tumor stage using the five-lncRNA signature (**A**) The Kaplan-Meier plot of the early stage patients with ccRCC (n=259). (**B**) The Kaplan-Meier plot of the late stage patients with ccRCC (n=181). (**C**) The Kaplan-Meier plot of the entire patients with ccRCC (n=440). The number below the curve represent the number of the patients in the high- and low-risk group. The *P-value* represents the differences between the two curves from the results of two-sided log-rank tests.

**Figure 5:**
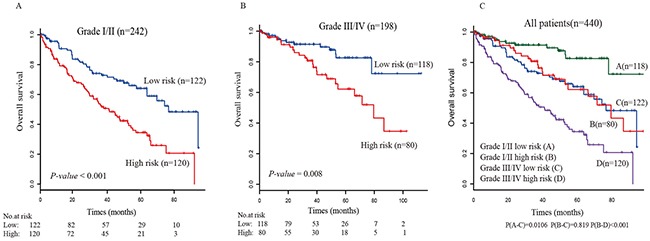
Stratification analyses of all patients adjusted to the tumor grade using the five-lncRNA signature (**A**) The Kaplan-Meier plot of the tumor grade I/II patients with ccRCC (n=259). (**B**) The Kaplan-Meier plot of the tumor grade III/IV patients with ccRCC (n=181). (**C**) The Kaplan-Meier plot of the entire patients with ccRCC (n=440). The number below the curve represent the number of the patients in the high- and low-risk group. The *P-value* represents the differences between the two curves from the results of two-sided log-rank tests.

We evaluated whether the survival prediction based on five-lncRNA signature was independent of clinical factors. Multivariate Cox regression analysis was then performed, including lncRNA-based risk score and other clinical information, such as age, gender, tumor grade and AJCC tumor stage (Table 2). The result showed that five-lncRNA risk score remained to be tightly associated with survival after adjusting the clinical factors. Moreover, we found that the age and AJCC stage were also significantly associated with overall survival. Then, stratified analysis was carried out, and the entire TCGA data set were divided into younger stratum (age ≤ 50, n=85) and older stratum (age > 50, n=355). The result showed that the five-lncRNA risk score can further divide ccRCC patients into high-risk and low-risk subgroup within each stratum (Figure 3). These result suggested that prognostic value of five-lncRNA signature is independent of age. Similar results were obtained when the stratification analysis of AJCC tumor stage (Figure 4) and tumor grade (Figure 5) were performed. These findings suggested that five-lncRNA risk score has a competitive performance for the survival prediction of ccRCC patients.

### Functional characteristics of five prognostic lncRNAs

**Figure 6:**
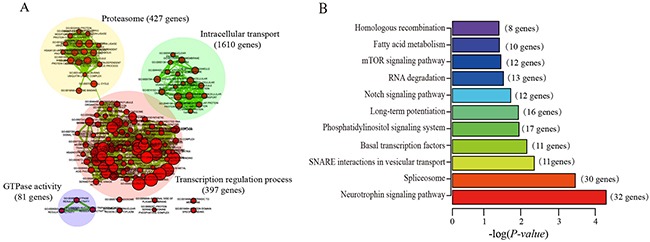
The results of functional enrichment analysis of the five lncRNA co-expressed protein-coding genes (**A**) The functional enrichment map of the GO terms. The nodes represent the enriched gene with the similarity functions. (**B**) KEGG pathways significantly associated with the co-expressed protein-coding genes.

To explore the functional implication of five prognostic lncRNAs in ccRCC tumorigenesis, we performed functional category enrichment analysis to examine their function. The biological functions of lncRNAs are still largely unknown. Many lncRNAs can act as *cis*-regulators, and the expression of lncRNA is significantly correlated with their neighboring protein-coding genes. Here, we predicted their putative functions based on co-expression network. Spearman correlation coefficients were calculated between lncRNAs and protein-coding genes based on their expression values. The top 1% protein-coding genes were selected as co-expressed partner of five prognostic lncRNAs. At last, a total of 1960 protein-coding genes were significantly correlated with at least one prognostic lncRNAs. Functional enrichment analysis showed that lncRNA correlated protein-coding genes were significantly enriched in 128 GO terms and 11 KEGG pathways (Figure 6). The functional categories are mainly involved in four functional clusters, including proteasome, transcription regulation process, intracellular transport, GTPase activity and several pathways (Figure 6A). This results suggested that the five prognostic lncRNAs might be involved in tumorigenesis process through regulating protein-coding genes to influence known cancer related pathways.

## DISCUSSION

Considering the great importance of lncRNAs in tumor tumorigenesis and progression, lncRNA dysregulation may serve as an important indicator of the characteristics of tumors. It has been documented that altered lncRNAs can exist in many cancer types, and are tightly associated with the outcome of cancers [32, 33]. Many works have focused on whether aberrant expression of specific lncRNAs in cancers can serve as independent markers for diagnosis and prognosis [17, 18, 31, 34]. Recently, although some works have announced the great importance of lncRNA in ccRCC tumorigenesis [23, 35], the comprehensive prognostic values of lncRNA in ccRCC have not been clarified clearly [36]. Therefore, a reliable prognostic biomarker is quite necessary.

In the present work, a five-lncRNA prognostic signature was identified based on the lncRNA expression profiles of ccRCC patients. It was then confirmed to be an independent prognostic predictor for patients with ccRCC. This study determined the potential five-lncRNA signature to predict the prognosis of ccRCC. The performance of five-lncRNA signature was evaluated using ROC analysis, suggesting that the prognostic value of the five-lncRNA signature is competitive for survival prediction. To the best of our knowledge, these five lncRNAs have not been previously reported, and further functional annotation of these prognostic lncRNAs will increase our understanding of their biological implications in determining ccRCC prognosis.

The result suggested that the prognostic value of five-lncRNA signature was independent of other clinical factors in ccRCC. Actually, lncRNAs have been reported to have higher specificity than mRNA in some cancer types [30, 37, 38]. The present work may bring some clinical implications in the development of novel prognostic factors in ccRCC. Although these five prognostic lncRNAs have not been previously investigated in cancers, we speculate that these lncRNAs may be involved in ccRCC tumorigenesis and many works are needed in the future ccRCC studies. Previous works have reported some prognostic lncRNAs in ccRCC, such as *TUG1* [39], *TCL6* [40], *H19* [41], *MALAT-1* [42] and *NBAT1* [35]. After measuring the prognosis of these lncRNAs using TCGA data in ccRCC, these lncRNAs are not involved in TCGA-based prognostic lncRNAs. We speculated the reasons why these reported prognostic lncRNAs cannot be validated in TCGA data. First, all these reported works are based on Chinese ccRCC patients, whereas the ccRCC patients in TCGA are Caucasian people, and the underlying molecular mechanisms might be different between populations. Second, these published works are all based on a small-scale ccRCC cases, which might draw a conclusion with deviation. Future works with more samples are necessary.

Several limitations of present work should be addressed. First, we only analyzed and validated the prognostic power of the five-lncRNA signature in the TCGA dataset, and no other ccRCC lncRNA expression data can be used for further validation. Although previously published microarray data can be used to identify some lncRNAs, these data only include a relatively small fraction of lncRNAs. Second, lncRNAs always play important regulatory roles in a wide range of biological processes through a complex regulatory network involving different kinds of *cis*- and *trans*-regulatory elements. Further integrated analysis may help us to predict the functional roles of the five prognostic lncRNAs in ccRCC more accurately. Third, no experimental data on the underlying mechanisms of lncRNAs have been performed, and future experimental studies on these lncRNAs can enhance our understanding of the functional role in ccRCC.

In this work, we reported a lncRNA signature in ccRCC patients to predict survival. Using large-scale independent expression profiles, we have demonstrated the prognostic values of lncRNAs in ccRCC patients. Our result has suggested that the five-lncRNA signature is helpful in predicting the clinical outcome, and may be an effective prognostic biomarker in the prediction of the survival of ccRCC patients.

## MATERIALS AND METHODS

### The ccRCC patient information

The lncRNAs expression data and corresponding clinical information of ccRCC patients TCGA database. After excluding the data without complete survival information, a total of 440 ccRCC patients were enrolled in this work. We also downloaded the detailed clinical information of ccRCC patients, including age, gender, tumor grade, AJCC cancer stage, etc. Samples from TCGA data set were equally divided into training (n=220) and testing sets (n=220).

### lncRNA expression profile

ccRCC RNA-seq data were downloaded from TCGA data portal (https://tcga-data.nci.nih.gov/tcga/). After alignment to the human genome (Ensembl database v72 assembly), the expression level of lncRNAs and mRNAs were determined by the value of Reads Per Kilobase of exon model per Million mapped reads (RPKM). We identified lncRNAs from TCGA dataset based on the following three criteria: 1) transcripts were not identified in any protein-coding region; 2) transcript sequences have been annotated in GENCODE project [7]; 3) transcripts were expressed in at least half of the ccRCC samples. The lncRNA expression profiles were defined as those with an average RPKM ≥ 0.1 across 440 ccRCC samples. At last, a total of 9669 lncRNAs in dataset were enrolled. We used edgeR [43] software to examine the expression difference.

### Statistical analysis

Based on the training set, the association between the expression level of each lncRNA and patient's overall survival was calculated using a univariate Cox regression. Those lncRNAs were considered to be significant if their *P-values* were less than 0.001. Then, the selected lncRNAs were fitted in a multivariate Cox regression analysis in the training dataset. Risk scores were estimated by involving these selected lncRNAs, which were weighted by their estimated regression coefficients in the multivariable Cox regression model. The risk score can be calculated for each ccRCC patient based on prognostic five-lncRNA signature. Based on the risk score formula, ccRCC patients can be divided into high-risk and low-risk groups, respectively. Differences in patients’ survival between these two groups can be evaluated by the Kaplan-Meier survival analyses. To further determine whether the prediction of the lncRNA signature was independent of other clinical variables, multivariate Cox regression and stratified analyses were carried out. The receiver operating characteristic (ROC) curve within 5 years were performed to compare the sensitivity and specificity of the survival prediction based on the risk score. All analyses were performed using R package (version 3.3.0).

### Functional enrichment analyses

To evaluate the functional implication of lncRNAs, spearman correlation coefficients were computed between five lncRNAs and protein-coding genes. Functional enrichment analyses for those co-expressed protein-coding genes were performed using the DAVID Bioinformatics Tool (version 6.7) [44]. GO and KEGG category enrichments were based on the threshold of *P-value* < 0.05 and enrichment score > 1.0. Significant enrichment results were visualized using Cytoscape software (version 3.4.0) [45].
